# Evolving frontiers in bladder cancer immunotherapy: integrating BCG, immune checkpoints, viral vectors, nanotechnology, and CAR-based therapies

**DOI:** 10.3389/fcell.2025.1719978

**Published:** 2025-12-04

**Authors:** Anna Di Spirito, Gaia Zuccolotto, Anna Tosi, Sahar Balkhi, Antonio Rosato, Denisa Baci, Lorenzo Mortara

**Affiliations:** 1 Immunology and General Pathology Laboratory, Department of Biotechnology and Life Sciences, University of Insubria, Varese, Italy; 2 Immunology and Molecular Oncology Unit, Veneto Institute of Oncology IOV - IRCCS, Padova, Italy; 3 Department of Immunology, Genetics and Pathology, Science for Life Laboratory, Uppsala University, Uppsala, Sweden; 4 Department of Surgery, Oncology and Gastroenterology, University of Padova, Padova, Italy; 5 Laboratory of Molecular Cardiology Laboratory, IRCCS-Policlinico San Donato, Milan, Italy

**Keywords:** bladder cancer, immunotherapy, BCG, immune checkpoint inhibitors, viral vector therapy, CAR platforms

## Abstract

Bladder cancer (BC) remains a prevalent malignancy with high recurrence rates despite standard therapies. Bacille Calmette-Guérin (BCG) is the cornerstone of treatment for non-muscle-invasive bladder cancer (NMIBC); however, nearly half of patients experience relapse or develop resistance, highlighting the need for alternative strategies. Recent advances in immunotherapy have reshaped the therapeutic landscape. Immune checkpoint inhibitors (ICIs) restore T-cell function and show clinical activity in BCG-unresponsive disease. Viral vector–based approaches, including nadofaragene firadenovec and CG0070, provide localized immune activation, while cellular platforms such as CAR-T and CAR-NK therapies offer precision targeting of tumor antigens. Concurrently, nanotechnology-based delivery systems and antibody–drug conjugates (ADCs) enhance efficacy and safety by improving tumor-specific cytotoxicity. Collectively, these strategies signify a paradigm shift from traditional intravesical therapy toward personalized and durable immunotherapeutic interventions. Identification of predictive biomarkers and rational combination strategies will be critical to improving outcomes and guiding the future management of BC.

## 1 Introduction

Bladder cancer (BC) is the 10th most common malignancy worldwide, affecting over 500,000 patients annually (Yamane et al., 2024). It predominantly affects men, although women often present a worse prognosis, partly due to diagnostic delays (Cohn et al., 2014). Environmental and genetic factors contribute to BC development; notable environmental risks include tobacco smoking and occupational exposures such as rubber manufacturing and firefighting (Di Spirito et al., 2025).

The tumor microenvironment (TME) plays a central role in BC progression and therapy resistance. It comprises a dynamic network of immune cells, fibroblasts, endothelial cells, extracellular matrix proteins, and signaling molecules that interact with tumor cells to promote growth, immune evasion, and therapeutic refractoriness (Di Spirito et al., 2025; Giraldo et al., 2019; Albini et al., 2018; Bruno et al., 2014).

Clinically and pathologically, BC is subdivided into two main categories: non-muscle-invasive (NMIBC) and muscle-invasive bladder cancer (MIBC) according to the depth of tumour invasion into the bladder wall (Flaig et al., 2024). At initial diagnosis, ∼75% of cases are NMIBC, whereas ∼25% are MIBC or metastatic (Kamat et al., 2016).

In NMIBC, tumour growth is limited to the urothelium or lamina propria but does *not* invade the muscularis propria (detrusor muscle). Specifically, NMIBC includes stages Ta (papillary tumour confined to the mucosa), Tis (carcinoma *in situ*, flat high-grade confined to mucosa) and T1 (tumour invades lamina propria) according to the TNM classification (Grabe-Heyne et al., 2023). By contrast, MIBC is defined as tumour invasion into the muscular layer (T2), and/or growth beyond into the perivesical tissue or adjacent organs such as in T3/T4 disease (Powles et al., 2022). Pathological grade is also a key differentiator: NMIBC may present as either low-grade or high-grade disease depending on cellular atypia and mitotic activity, while MIBC is typically high-grade by definition, reflecting its aggressive behaviour and higher risk of progression.

Additional clinical criteria such as tumour size, number of lesions, presence of carcinoma *in situ* (CIS), and prior recurrence also influence risk stratification and management decisions for NMIBC (Grabe-Heyne et al., 2023).

NMIBC is primarily managed with transurethral resection of bladder tumor (TURBT) followed by intravesical instillations of mitomycin C or BCG. MIBC is treated with radical cystectomy (RC) combined with neoadjuvant platinum-based chemotherapy (Yang et al., 2024). RC carries high perioperative risk, particularly in elderly patients with comorbidities (Ramani et al., 2010). Despite BCG being the standard immunotherapy for NMIBC, limitations in efficacy, toxicity, and global supply shortages necessitate the development of novel therapeutic strategies.

In recent years, immunotherapy has garnered significant attention as a novel therapeutic approach in BC. Different types of immunotherapy, including immune checkpoint inhibitors (ICIs), vaccines, and adoptive cell therapies, are under investigation. These approaches are summarized in Table 1 and discussed in detail below.

**TABLE 1:** Evolution and frontiers of immunotherapy in bladder cancer.

Therapeutic approach	Mechanism of action	Key clinical evidence	Advantages	Limitations and challenges
BCG (Bacillus Calmette–Guérin)	• Acts as PAMP → activates PRRs (e.g., TLRs) on urothelial and immune cells • Induces cytokine release (IL-6, IL-8, TNF-α) → recruits neutrophils, DCs, T cells, NK cells • Promotes Th1 polarization, CD8^+^ cytotoxicity, NK/macrophage activity • Direct cytotoxic effects via TRAIL, perforin, caspase pathways	• Morales et al. (1976): First efficacy report • Sylvester meta-analysis: 24 trials, >4,800 patients	• Proven efficacy in high-risk NMIBC. • Gold standard for decades • Enhances both innate and adaptive immunity	• Failure rate: 40%–50% • Local/systemic toxicities • Frequent relapse • Global shortages • Resistance via PD-L1 upregulation, T-cell exhaustion, chronic inflammation
Checkpoint inhibitors (ICIs)	Block inhibitory signaling – PD-1/PD-L1 axis → restores T-cell cytotoxicity	• KEYNOTE-057: Pembrolizumab in BCG-unresponsive NMIBC, CR 41% in CIS patients → FDA approval (2020) • KEYNOTE-676 and CheckMate 9UT: evaluating ICI + BCG.	• Effective in BCG-unresponsive patients • Durable responses in subsets • Expanding role in NMIBC and MIBC.	• Limited response in “cold” TME. • Immune-related toxicities • High cost
Oncolytic and gene therapy vectors	• CG0070: Selectively replicates in Rb-deficient cells, expresses GM-CSF → immune activation • Adstiladrin: Non-replicating adenovirus delivering IFN-α2b gene → cytokine production, NK activation	• CG0070: Phase I showed safety and efficacy in BCG-refractory NMIBC; NCT04387461 testing CG0070 + pembrolizumab • Adstiladrin: Phase III → CR 51% at 3 months, ∼50% durable at 12 months. FDA-approved (first adenoviral gene therapy in BC)	• Localized intravesical action • Strong local immune response • Favorable safety profile	• Durability still limited • Viral delivery challenges • Patient selection optimization needed
CAR-T cell therapy	T cells engineered with CARs targeting tumor antigens (e.g., SIA-CIgG) → tumor killing via perforin/granzyme	• Preclinical: CAR-T against SIA-CIgG effective; synergy with vorinostat enhanced efficacy	• Highly specific cytotoxicity • Potential long-term persistence	• Antigen heterogeneity • Poor solid tumor infiltration • Risk of CRS/neurotoxicity • Complex, individualized manufacturing
CAR-NK cell therapy	NK cells engineered with CARs (e.g., CD24-targeted) → cytotoxic granules, ADCC-mediated killing	• Preclinical: CAR-NK against CD24 effective in urothelial tumors	• “Off-the-shelf” potential • Lower CRS and GvHD risk • Shorter lifespan reduces chronic toxicity	• Limited persistence *in vivo* • Still early in clinical development
Nanoformulation-based immunotherapy	Nanoparticles improve intravesical delivery, adhesion, and penetration Examples: Chitosan/lipid nanoparticles for BCG. – Exosome-mimetic vesicles (CD73 inhibitor + anti-PD-L1)	• EMV-CD73/anti-PD-L1: enhanced CTL activity, tumor growth reduction in mice	• Improves drug retention and safety • Enables combination strategies (e.g., immunotherapy + targeted delivery)	• Mostly preclinical stage • Bladder permeability barrier remains challenging
Antibody–Drug conjugates (ADCs)	Antibodies linked to cytotoxic agents	• Enfortumab vedotin (Nectin-4) and sacituzumab govitecan (TROP-2) → FDA-approved for advanced BC.	• High tumor selectivity • Synergy with ICIs • Expanding options for advanced BC.	• Toxicities (skin, neuropathy, diarrhea) • Resistance potential • High cost and limited access

### 1.1 Immunotherapy in bladder cancer: the BCG legacy

BC appears particularly receptive to immunotherapeutic strategies due to its high baseline immune gene expression and the upregulation of checkpoint molecules in the tumor milieu (Lenfant and Rouprêt, 2018; Nair et al., 2020). BCG, a live attenuated strain of *Mycobacterium bovis*, was initially developed as a tuberculosis vaccine by Calmette and Guérin in 1921 (Herr and Morales, 2008). Its antitumor potential was recognized in the 1950s, and Morales et al. first demonstrated clinical efficacy for superficial bladder tumors in 1976 (Morales et al., 1976). Since then, BCG has remained the most effective local therapy for NMIBC (Böhle and Brandau, 2003; Brandau and Suttmann, 2007; Liu et al., 2022).

### 1.2 Immune mechanism of BCG

**FIGURE 1:**
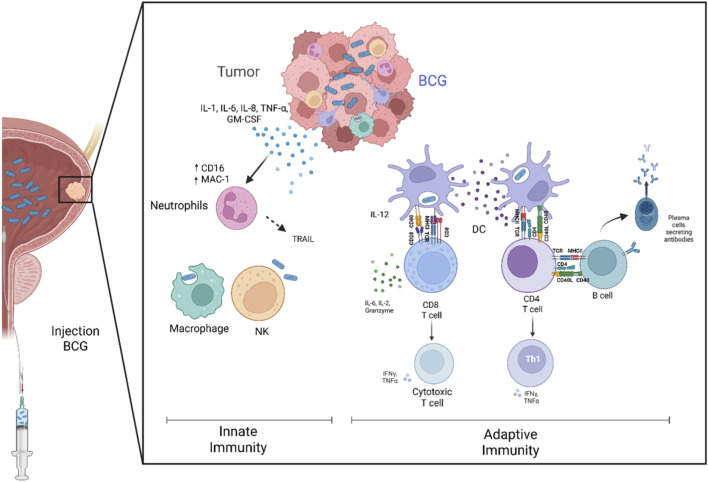
Mechanism of BCG intravesical therapy in bladder cancer. Following instillation, BCG adheres to and is internalized by urothelial and immune cells, activating pathogen recognition receptors (PRRs) and triggering the release of proinflammatory cytokines (IL-6, IL-8, IL-17, TNF-α) and chemokines. This immunogenic microenvironment recruit neutrophils, macrophages, and dendritic cells: neutrophils induce tumor apoptosis via TRAIL and NETs, while DCs mature, migrate to lymph nodes, and activate tumor and BCG specific T cells (Th1 and cytotoxic). In addition to cell-mediated immunity, BCG exerts direct cytotoxic effects through apoptosis (TRAIL, perforin, caspases) and the induction of autophagy, enhancing malignant cell clearance and reducing recurrence.

BCG acts as a pathogen-associated molecular pattern (PAMP), activating pattern recognition receptors (PRRs) such as Toll-like receptors (TLRs) on urothelial and antigen-presenting cells. After intravesical instillation, BCG adheres to the urothelium and is internalized by both normal and malignant urothelial cells as well as resident immune cells. This uptake triggers the release of pro-inflammatory cytokines (IL-6, IL-8, IL-17, TNF-α) and chemokines, generating an immunogenic microenvironment that recruits circulating immune cells (Figure 1).

Neutrophils are the first responders, attracted mainly by IL-8 and IL-17. Their presence in urine correlates with favorable outcomes, as neutrophils induce tumor cell apoptosis through tumor necrosis factor-related apoptosis-inducing ligand (TRAIL) release and neutrophil extracellular traps (NETs). Dendritic cells (DCs) then play a pivotal role in bridging innate and adaptive immunity. Upon BCG stimulation, DCs upregulate costimulatory molecules (CD40, CD80, CD86, MHC-II), secrete IL-12 to drive Th1 polarization, and migrate to lymph nodes to present BCG and tumor antigens, activating naïve T cells.

In addition to immune-mediated killing, BCG exerts direct cytotoxicity. It induces apoptosis through TRAIL, perforin, pro-caspase-9, BID activation, and cathepsin B upregulation, as well as caspase-independent pathways involving HMGB1 (Sandes et al., 2007; See et al., 2009). Furthermore, BCG can promote autophagy in tumor cells, amplifying its anti-tumor effects (Tavakoly et al., 2018; Kleinnijenhuis et al., 2012).

In summary, BCG immunotherapy transforms the bladder into a localized immunogenic site, where coordinated innate and adaptive immune mechanisms, supported by direct cytotoxic effects, eliminate malignant cells and reduce recurrence.

### 1.3 Efficacy in NMIBC and failure rates

Multiple randomized trials and meta-analyses have demonstrated that BCG significantly reduces recurrence rates in NMIBC compared with TURBT alone or with intravesical chemotherapy. Sylvester et al.’s meta-analysis of 24 trials (including over 4,800 patients) reported a 32% reduction in the risk of recurrence with BCG maintenance therapy with particular benefit in high-risk patients (multifocal tumors, carcinoma *in situ* (CIS), or high-grade disease) (Sylvester, 2011). Moreover, two meta-analyses found that BCG therapy may also reduce the risk of tumor progression (Böhle and Bock, 2004; Sylvester et al., 2002), and BCG maintenance therapy appears to be significantly better in preventing recurrences than chemotherapy (Malmström et al., 2009; Sylvester et al., 2010), albeit with more side effects (Shang et al., 2011). Complete response after BCG instillations can be achieved in up to 70% of patients, but unfortunately around 40%–50% will experience recurrence or progression within 5 years (Zlotta et al., 2009). Additionally, not all patients can complete the treatment course due to local or systemic side effects (Lamm et al., 2000), including dysuria, hematuria, fever, and nausea. Although less common, systemic side effects are often serious and potentially life-threatening. Although rare, serious systemic complications can occur, often due to BCG sepsis from traumatic catheterization or premature post-TURBT instillation (Lamm, 1992).

Failure of BCG can be attributed to several factors. Many patients display insufficient or dysregulated host immune response, heterogeneous tumor antigen expression or, other tumor immune evasion mechanisms. Furthermore, global shortages of BCG, particularly since the early 2010s have forced many centers to ration doses or seek alternative regimens. Thus, there is a significant urgent need for novel biomarkers to predict BCG response and for alternative strategies for patients who do not respond to BCG.

### 1.4 Causes of BCG resistance

One principal mechanism of tumor-mediated immune escape in BCG therapy is the upregulation of PD-L1 on tumor cells and antigen-presenting cells. The interaction between PD-L1 and the PD-1 receptor on activated T cells initiates inhibitory signaling cascades that suppress T-cell proliferation, cytokine production, and cytotoxic function, leading to exhausted CD8^+^ T cells with impaired tumor-killing capacity. Importantly, BCG therapy can induce the release of inflammatory molecules such as IL-10 and activate the STAT3 pathway, which in turn enhances PD-L1 expression and diminishes the adaptive immune response creating a negative feedback loop of immunosuppression in some patients.

Lim et al. demonstrated that TME of BCG responders was enriched with active CD8^+^ T cells, lacking PD-L1 expression and with FoxP3-negative CD4^+^ T cells, while the TME of non-responders was enriched with exhausted PD-L1^+^ CD8^+^ T cells (Lim et al., 2021). Similarly, Kates et al. identified an absence of baseline CD4^+^ T cells in PD-L1^+^ non-responders, indicating another potential mechanism of BCG resistance (Kates et al., 2020).

Inefficient trafficking of CD4^+^ T cells into the TME may also contribute to resistance, potentially arising from downregulation of adhesion molecules on endothelial cells or mismatched expression of chemokine receptors, including CXCR3, CXCL9, and CXCL10 (Pichler et al., 2016).

These findings underscore the therapeutic potential of immune checkpoint blockade to restore BCG-induced T-cell activity, a strategy currently being evaluated in prospective clinical trials (Meghani et al., 2022; Giannarini et al., 2022).

Additionally, repeated BCG exposure can lead to chronic inflammation, promoting regulatory immune pathways, tumor resistance mechanisms, and progressive T-cell exhaustion. Consequently, the TME becomes increasingly refractory to immune surveillance, despite the presence of activated effector cells. Inter-patient variability in BCG strain virulence and intrinsic immune competence further contributes to heterogeneous clinical responses.

This provides a compelling rationale for combining BCG with anti-PD-L1 agents to potentially overcome treatment failure and improve outcomes.

## 2 Modern immunotherapeutic approaches

Recent advances in BC therapy have focused on immunotherapeutic strategies to address the shortcomings of BCG. These “next-generation” approaches aim to restore antitumor immunity and remodel the TME through various mechanisms. Notably, ICIs and oncolytic viral therapies have emerged as promising modalities, aiming to restore antitumor immunity and remodel the TME.

### 2.1 Immune checkpoint inhibitors

ICIs have fundamentally reshaped the therapeutic landscape of BC by targeting inhibitory pathways that restrain T-cell–mediated antitumor activity (Peggs et al., 2009). The most extensively studied checkpoints in BC are the PD-1/PD-L1 axis and CTLA-4. PD-1, expressed on T cells, binds PD-L1 (and PD-L2) on tumor or immune cells to dampen immune responses.

Anti-PD-1 agents, such as pembrolizumab, block PD-1 interactions with both PD-L1 and PD-L2, potentially providing a broader reactivation of antitumor immunity, whereas anti-PD-L1 therapies selectively inhibit PD-L1 engagement with PD-1 while leaving PD-L2 signaling intact. CTLA-4 is another checkpoint that negatively regulates T-cell activation at an earlier stage within lymphoid tissues; its blockade releases inhibitory signals during the T-cell priming phase, in contrast to the peripheral re-invigoration achieved through PD-1/PD-L1 inhibition (Zhang et al., 2019; Abaza et al., 2023).

In the context of BCG-unresponsive NMIBC checkpoint inhibitors have shown clinical efficacy. In the single-arm KEYNOTE-057 trial pembrolizumab monotherapy achieved a complete response rate of 41% at 3 months in patients with CIS who were ineligible for or refused cystectomy (Cohort A, n = 96), with durable responses and a manageable toxicity profile, leading to FDA accelerated approval in January 2020 (Balar et al., 2021). More recently, in Cohort B, which enrolled patients with high-grade papillary tumors without CIS, pembrolizumab yielded a 12-month disease-free survival of 43.5%, further underscoring its therapeutic relevance across diverse NMIBC subtypes (Necchi et al., 2024).

Ongoing studies continue to expand the scope of ICIs in BC. For instance, The CheckMate 9UT trial evaluates nivolumab alone or with the IDO-1 inhibitor BMS-986205 and with or without BCG, in patients with BCG-unresponsive CIS (Albisinni et al., 2021).

Additionally, novel checkpoints are being investigated: agents targeting TIGIT (e.g., Vibostolimab) and LAG-3 (e.g., Favezelimab) are now in clinical trials combined with pembrolizumab for BCG-unresponsive disease, reflecting an effort to reverse immune exhaustion by multiple mechanisms (Kulkarni et al., 2024). In patients with more advanced BC, ICIs are already standard in certain settings (for example, avelumab maintenance therapy after chemotherapy in metastatic disease, and combination immunotherapy with chemotherapy in frontline metastatic urothelial carcinoma) proving the principle that unleashing anti-tumor T cell activity can improve outcomes even beyond NMIBC (Gupta et al., 2025; Powles et al., 2020).

Collectively, checkpoint blockade strategies aim to overcome tumor-induced immunosuppression (such as that which may underlie BCG failure) and offer more effective, bladder-sparing treatment options for high-risk patients.

### 2.2 Viral vectors therapies

The use of engineered viral vectors is a promising strategy in BC treatment, with the advantage of enabling localized immune effects within the bladder (Wang et al., 2023).

Two therapies based on adenoviruses have been subjected to clinical trials. CG0070, a genetically engineered oncolytic adenovirus was designed to selectively replicate in BC cells with defects in the retinoblastoma (Rb) tumor suppressor pathway, and concurrently produce granulocyte-macrophage colony-stimulating factor (GM-CSF) to stimulate immune-mediated anti-tumor effects (Burke et al., 2012).It uses the human E2F-1 promoter, which is active in cells with a compromised Rb pathway, to drive the expression of the key viral gene E1A, thereby ensuring that viral replication occurs selectively in tumor cells lacking functional Rb. The virus additionally contains the gene for human GM-CSF, which is situated beneath the E3 promoter of the adenovirus. Given that the E3 promoter is stimulated by E1A, GM-CSF is predominantly generated in tumor cells where viral replication takes place.

A first-in-human Phase I trial demonstrated that CG0070 can safely induce meaningful clinical responses in patients with BCG-refractory NMIBC.

Moreover, the Phase II CORE-001 study (NCT04387461), evaluating intravesical CG0070 in combination with systemic pembrolizumab in patients with BCG-unresponsive NMIBC, reported high complete response rates at early time points and encouraging durability. Investigators observed response rates substantially higher than those historically achieved with checkpoint inhibitor monotherapy (pembrolizumab), in a phase 2 study (Necchi et al., 2024), while maintaining a toxicity profile comparable to that of the individual agents. This combination may therefore improve the benefit–risk ratio for patients with BCG-unresponsive carcinoma *in situ* (Li et al., 2024).

Nadofaragene Firadenovec, also known as Adstiladrin, is a non-replicating recombinant adenoviral vector therapy that delivers the human interferon-α2b (IFNα2b) gene into bladder urothelial cells, transforming them into local cytokine-producing “micro-factories” (Steinmetz et al., 2024).

This approach harnesses the potent anti-tumor and immunomodulatory functions of Type I interferon, including NK cell activation and anti-angiogenic effects, providing a targeted intravesical immunotherapeutic strategy for BCG-unresponsive NMIBC.

Adstiladrin’s clinical development has demonstrated both efficacy and durability in BCG-unresponsive NMIBC. The pivotal Phase III, open-label, single-arm trial (NCT02773849) evaluated intravesical nadofaragene firadenovec in 157 patients with high-grade, BCG-unresponsive NMIBC, including 103 patients with carcinoma *in situ* (CIS) ± Ta/T1 and 48 patients with high-grade Ta/T1 without CIS (Boorjian et al., 2021; ClinicalTrials.gov, 2024).

Within the CIS ± Ta/T1 cohort, the CR rate was recorded at 53.4% within 3 months following the initial instillation, with a median CR duration of 9.7 months and 45.5% of responders maintaining a high-grade recurrence-free status at 12 months.

For the high-grade Ta/T1 papillary cohort (n = 48), 72.9% of patients were high-grade recurrence-free at 3 months, and 43.8% at 12 months, with a median high-grade recurrence-free survival of 12.4 months.

Progression to muscle-invasive disease was observed in approximately 5%–6% of patients.

The therapy exhibited a favorable safety profile, with most treatment-related adverse events being grade 1–2 (local bladder irritation, fatigue, and urinary frequency); only 4% of patients experienced grade 3–4 events, and no grade 5 events were drug-related (Boorjian et al., 2021).

Based on these results, Adstiladrin became the first FDA-approved adenoviral vector-based gene therapy for BC in December 2022, representing a novel and effective therapeutic option for patients with BCG-unresponsive NMIBC (Colbert et al., 2025).

## 3 Cellular immunotherapies

Cell-based immunotherapy is an emerging frontier in BC treatment. Chimeric antigen receptor T-cell (CAR-T) therapy involves engineering a patient’s own T lymphocytes to recognize a specific tumor-associated antigen, enabling precise and potent immune responses against tumor cells. Similarly, CAR-NK cell therapy modifies natural killer cells (from the patient, a donor, or an established cell line) with CARs to enhance their innate tumor-killing capacity. CAR-NK cells may offer some safety and logistical advantages over CAR-T, including a lower risk of graft-versus-host disease and cytokine release syndrome, and potential for “off-the-shelf” use due to NK cells’ short lifespan and HLA-independent targeting.

### 3.1 CAR-T and CAR-NK

Recent studies have developed CAR-T cells specifically targeting SIA-CIgG, a sialylated form of immunoglobulin G overexpressed in BC cells. These CAR-T cells demonstrated potent tumor cell lysis *in vitro* and improved persistence compared with HER2-directed CAR-T cells. Furthermore, combining these CAR-T cells with epigenetic modulators such as the HDAC inhibitor vorinostat) enhanced their antitumor activity, suggesting a potential path to improve efficacy (Ding et al., 2024).

In parallel, several other CAR-T strategies have shown promise in preclinical BC models. Parriott et al. (Parriott et al., 2020) demonstrated that chimeric PD-1 T cells induce potent tumor lysis and durable tumor-free survival in syngeneic BC mouse models, underscoring the potential of checkpoint-targeted CAR-T approaches. MUC1-CAR-T cells exhibited selective cytotoxicity against MUC1-positive BC organoids (Yu et al., 2021), while combining decitabine with EGFR- or CD44v6-CAR-T cells enhanced cytotoxicity against urothelial carcinoma cell lines (Grunewald et al., 2021). HER2 remains an appealing, though debated, target (Yan et al., 2015). Nectin-4, already validated as a therapeutic target through the antibody–drug conjugate enfortumab vedotin, approved for advanced urothelial carcinoma (Heath and Rosenberg, 2021) has further spurred the development of NECTIN4-directed CAR-T cells. NECTIN4-directed CAR-T cells demonstrated potent cytotoxicity across urothelial carcinoma cells expressing varying levels of endogenous NECTIN4, including those resistant to enfortumab vedotin. Moreover, activation of the PPARγ pathway with the agonist rosiglitazone upregulated NECTIN4 expression, further enhancing CAR-T efficacy, suggesting a rational combination strategy for EV-refractory patients (Chang et al., 2025) and additional antigens such as cancer-restricted glycosaminoglycan (Khazamipour et al., 2024) and PSCA engineered to overcome CD155-mediated inhibition (Shen et al., 2024) have also shown therapeutic benefit. Moreover, B7H3-CAR-T cells demonstrated efficacy in BC organoids, supporting their use in precision immunotherapy (Jiang et al., 2023). Together, these findings highlight a growing spectrum of antigen-specific CAR-T approaches that may complement existing immunotherapies for advanced BC (Supplementary Table S1).

Similarly, CAR-NK cell therapy modifies natural killer (NK) cells (from the patient, a donor, or established cell lines) with CAR constructs to enhance their innate tumor-killing capacity. NK cells engineered with CARs targeting CD24 have shown specific cytotoxic activity against various urologic tumors, including BC, confirming CD24 as a relevant therapeutic target (Söhngen et al., 2023). Compared with CAR-T cells, CAR-NK cells present several intrinsic advantages, including a lower risk of cytokine release syndrome and neurotoxicity, the absence of graft-versus-host disease, and the feasibility of “off-the-shelf” allogeneic use due to their short lifespan and HLA-independent recognition. These characteristics make CAR-NK cells particularly attractive for clinical translation, especially in localized settings such as BC, where intravesical administration could minimize systemic exposure and toxicity (Balkhi et al., 2025).

Despite these advantages, CAR-NK cells exhibit comparatively limited *in vivo* persistence and durability of response, partly due to their short lifespan and the immunosuppressive tumor microenvironment. To address these limitations, current engineering efforts focus on improving survival, metabolic fitness, and antitumor function. One promising approach involves incorporating cytokine support, such as IL-15, directly into the CAR construct, which enhances proliferation and longevity without systemic cytokine toxicity. Furthermore, induced pluripotent stem cell (iPSC)–derived NK platforms provide a renewable, standardized, and gene-editable source of effector cells with consistent potency, facilitating large-scale, off-the-shelf manufacturing. Additional strategies include feeder-based expansion systems expressing IL-21 or 4-1BBL to promote memory-like phenotypes, and combinatorial treatments with immune checkpoint inhibitors or cytokine agonists (such as N-803) to overcome local immunosuppression. Preclinical work also explores localized delivery systems, including hydrogels and nanomaterial scaffolds, to retain CAR-NK cells within the bladder wall and sustain their activity (Kumar et al., 2025; Van der Meer et al., 2021).

Overall, CAR-T cells currently demonstrate greater persistence and durability, while CAR-NK platforms offer superior safety, scalability, and potential for allogeneic use. Continuous innovations especially IL-15-engineered and iPSC-derived NK systems are rapidly closing the efficacy gap, paving the way for long-lasting, off-the-shelf cellular therapies that may transform the management of both NMIBC and MIBC in the coming years.

Efforts are also underway to improve tumor penetration and persistence of CAR-based therapies in solid tumors like BC.

Notably, efforts are underway to improve the tumor penetration and persistence of CAR-based therapies in solid tumors such as BC. One experimental approach explores the feasibility of localized CAR-T administration directly into the bladder. For instance, an early-phase clinical trial (NCT03185468) (ClinicalTrials.gov, 2025) is investigating fourth-generation CAR-T cell therapy in patients with locally advanced or metastatic urothelial carcinoma targeting prostate-specific membrane antigen (PSMA) and folate receptor-α (FRα), in a non-randomized, open-label Phase I/II design. This pivotal study primarily aims to evaluate the safety, tolerability, and feasibility of CAR-T therapy in urothelial carcinoma and conceptually supports the potential for localized CAR-T delivery in BC.

Such approaches could inform future trials employing bladder-specific antigens if intravesical delivery platforms prove safe.

Although CAR-based treatments for BC remain in early development, they represent an emerging and promising avenue for next-generation immunotherapy.

## 4 Nanoformulation-based strategies

Improving intravesical drug delivery is a critical strategy for bladder cancer, given the unique challenge posed by the bladder’s impermeable urothelial barrier. Nanoparticle-based delivery systems offer the potential to increase therapeutic penetration and retention in bladder tissues. Nanoparticles have a high surface-area-to-volume ratio and can be engineered for enhanced adhesion to the bladder wall, making them attractive vehicles for intravesical therapy (GuhaSarkar and Banerjee, 2010). Combining immunotherapy with nanoparticle-based delivery systems represents a promising strategy to improve efficacy and reduce toxicity (Winnicka et al., 2024).

Therapy with the BCG vaccine is one of the most common nanomedicine platform for BC (Erdoğar et al., 2015a). Erdoğar et al. (2015b) used cationic chitosan nanoparticles for the safe and effective intravesical delivery of BCG. This formulation enhanced antitumor efficacy, prolonged survival, and reduced systemic toxicity in preclinical models. Other scientists ameliorated this formulation for BCG therapy, using lipid nanoparticles, in order to enhance delivery to bladder wall (Nakamura et al., 2014).

Zhou et al. (2021) instead, designed macrophage-derived exosome-mimetic nanovesicles (EMVs) in order to deliver the CD73 inhibitor (AB680) and the monoclonal antibody (anti-PD-L1). *In vivo* studies in BC mice models, demonstrated a favorable biosafety profiles, stability, and especially specific delivery to BC. This combination enhanced activation of cytotoxic T-lymphocytes, and consequently a decrease of tumor growth and increased survival.

### 4.1 Antibody-drug conjugates

Antibody–drug conjugates (ADCs) are emerging as a transformative strategy in BC treatment. By combining the specificity of monoclonal antibodies with the cytotoxic potency of chemotherapeutics, ADCs can selectively target tumor cells while sparing normal tissues. Recent clinical evidence highlights their potential to complement or even synergize with ICIs, thereby expanding the therapeutic arms against advanced BC (Zhang and Li, 2025). Notable ADCs approved for BC include Enfortumab vedotin, targeting Nectin-4, and Sacituzumab govitecan, targeting TROP-2. These agents have shown promising efficacy in clinical trials, offering new hope for patients with advanced disease (Hanna et al., 2022; McGregor et al., 2023). Furthermore, a novel bispecific ADC targeting both EGFR and HER3, receptors implicated in urothelial carcinoma pathogenesis, has shown promising antitumor activity in a Phase I study across several tumor types, with a good overall response rate (Ma et al., 2024). By hitting two targets, such an approach might overcome tumor heterogeneity and resistance. While still investigational, these advances illustrate how ADC technology is rapidly evolving as part of the immunotherapy arsenal.

## 5 Future directions and conclusion

Bladder cancer therapy is undergoing a rapid evolution from the historic BCG era to a modern landscape enriched by ICIs, gene therapies, cellular immunotherapies, and precision delivery systems. Despite these advances, significant hurdles remain, including heterogeneous patient responses, immune escape mechanisms, treatment-related toxicities, and the absence of robust predictive biomarkers. Addressing these challenges will require a multi-pronged strategy.

First, the integration of biomarker-driven precision medicine is critical to guide therapeutic selection, optimize efficacy, and minimize unnecessary toxicity. Genomic, transcriptomic, and immunophenotypic profiling of tumors may enable us to predict which patients will respond to ICIs, oncolytic viruses, or cell therapies, and thus personalize treatment approaches. Second, the rational design of combination regimens holds great promise. Pairing cellular immunotherapies (e.g., CAR-T, CAR-NK) with checkpoint inhibitors or antibody–drug conjugates could overcome resistance by simultaneously targeting multiple immune-evasion pathways. Likewise, coupling nanotechnology-based delivery systems with established therapies may improve drug penetration, durability, and safety. In the future, we might see “triplet” regimens (e.g., virus + ICI + nanoparticle drug) or other innovative combinations to attack the tumor from all angles.

Third, advancing allogeneic and off-the-shelf platforms particularly CAR-NK therapies may expand access while reducing logistical barriers associated with autologous approaches. In parallel, continued innovation in intravesical delivery systems could maximize local tumor control, reduce the need for radical surgeries while sparing patients systemic toxicity.

Finally, prospective clinical trials with robust correlative studies are essential to validate emerging strategies and unravel mechanisms of response and resistance. As immunotherapy pipelines expand, long-term monitoring of safety, durability, and quality of life will remain equally important.

In conclusion, the therapeutic landscape of BC is rapidly evolving beyond conventional surgery and BCG. By combining novel immunotherapies, biomarker-guided personalization, and innovative delivery technologies, the field is moving toward a new era of more effective, durable, and patient-tailored treatment strategies.

## Abbreviations

ADCs, Antibody–drug conjugates; ADCC, Antibody-Dependent Cell-mediated Cytotoxicity; BC, Bladder cancer; BCG, *Bacillus* Calmette–Guérin; CAR, Chimeric antigen receptor; CTLs, Cytotoxic CD8^+^ T lymphocytes; DCs, Dendritic cells; FRα, Folate receptor-α; ICIs, Immune checkpoint inhibitors; MIBC, Muscle-invasive bladder cancer; NETs, Neutrophil extracellular traps; NMIBC, Non-muscle-invasive bladder cancer; PD-1, Programmed death one receptor; PD-L1, Programmed death ligand-1; PSMA, Prostate-specific membrane antigen; TME, Tumor microenvironment; TRAIL, Tumor necrosis factor-related apoptosis-inducing ligand; TURBT, Transurethral resection of bladder tumor.
