# Supplementary material for: Evolving frontiers in bladder cancer immunotherapy: integrating BCG, immune checkpoints, viral vectors, nanotechnology, and CAR-based therapies
Source: Front Cell Dev Biol. 2025 Dec 4;13:1719978. doi: 10.3389/fcell.2025.1719978 (PMC12711830; doi:10.3389/fcell.2025.1719978)
Supplement: Supplementary file 1 [file Table1.docx]

| Antigen | Biological role / relevance in bladder cancer | Status of CAR-T / CAR-NK development | Key references |
| --- | --- | --- | --- |
| NECTIN4 | Cell adhesion molecule highly expressed in urothelial carcinoma; target of approved ADC *enfortumab vedotin*. | Preclinical CAR-T constructs show strong in vitro and in vivo cytotoxicity against NECTIN4⁺ bladder cells. | Chang et al, 2025, Heath et al, 2021 |
| MUC1 | Aberrantly glycosylated mucin overexpressed in urothelial carcinoma; associated with tumor invasion and immune evasion. | Specific cytotoxicity against MUC1-positive BC organoids | Yu et al, 2021 |
| EGFR / HER2 | RTK family; variably expressed in urothelial carcinoma; involved in resistance and signaling. | 1. EGFR/CD44v6: Preclinical, Enhanced cytotoxicity when combined with decitabine 2. HER2: Overexpressed in BC, potential target for deeper investigation, currently recruits, (NCT03740256) | Grunewald et al, 2021 and Patelli et al, 2022 |
| PSCA(with TIGIT) | Prostate stem cell antigen; expressed in bladder carcinoma. | Enhanced efficacy by overcoming CD155-mediated inhibition, preclinical only. | Shen et al, 2024 |
| CD24 / SIA-CIgG | Emerging bladder-associated surface molecules linked to stemness and immune evasion. | Effective BC cell lysis with enhanced persistence and enhanced efficacy with vorinostat combination, preclinical only | Ding et al, 2024 |

Trials:

NCT03740256 is investigating HER2-specific autologous CAR-T cells with intratumoral OVs injection in mUC. NCT04660929 is evaluating an immuno-oncology treatment for solid tumors based on CAR macrophages (CAR-M) that were transduced with HER2-specific chimeric receptors.

Finally, NCT04319757 is testing a novel trastuzumab-conjugated NK cell therapy (ACE1702)

Chang K, Delavan HM, Yip E, Kasap C, Zhu J, Lodha R, Liao SY, Berman SC, Carretero-Gonzalez A, Basar M, Gokturk Ozcan G, Teo MY, Solit DB, Rosenberg JE, Al-Ahmadie H, Ding CCK, Chan E, Steri V, Porten SP, Koshkin VS, Friedlander TW, Feng FY, Lee JK, Wiita AP, Chu CE, Chou J. Modulating the PPARγ pathway upregulates NECTIN4 and enhances chimeric antigen receptor (CAR) T cell therapy in bladder cancer. Nat Commun. 2025 Sep 10;16(1):8215. doi: 10.1038/s41467-025-62710-0. PMID: 40931013; PMCID: PMC12423289.

Heath EI, Rosenberg JE. The biology and rationale of targeting nectin-4 in urothelial carcinoma. Nat Rev Urol 2021; 18:93–103.

Yu L, Li Z, Mei H, Li W, Chen D, Liu L, Zhang Z, Sun Y, Song F, Chen W, Huang W. Patient-derived organoids of bladder cancer recapitulate antigen expression profiles and serve as a personal evaluation model for CAR-T cells in vitro. Clin Transl Immunology. 2021 Jan 31;10(2):e1248. doi: 10.1002/cti2.1248. PMID: 33552510; PMCID: PMC7847802.

Grunewald CM, Haist C, König C, Petzsch P, Bister A, Nößner E, Wiek C, Scheckenbach K, Köhrer K, Niegisch G, Hanenberg H, Hoffmann MJ. Epigenetic Priming of Bladder Cancer Cells With Decitabine Increases Cytotoxicity of Human EGFR and CD44v6 CAR Engineered T-Cells. Front Immunol. 2021 Nov 17;12:782448. doi: 10.3389/fimmu.2021.782448. PMID: 34868059; PMCID: PMC8637820.

Patelli G, Zeppellini A, Spina F, Righetti E, Stabile S, Amatu A, Tosi F, Ghezzi S, Siena S, Sartore-Bianchi A. The evolving panorama of HER2-targeted treatments in metastatic urothelial cancer: A systematic review and future perspectives. Cancer Treat Rev. 2022 Mar;104:102351. doi: 10.1016/j.ctrv.2022.102351. Epub 2022 Jan 31. PMID: 35180563.

Shen JJ Dai DP Zhao WX, et al. A novel co-receptor with mutated TIGIT to enhance PSCA CAR-T therapy for bladder cancer. J Clin Oncol 2024;42(16):e14574. DOI: 10.1200/JCO.2024.42.16_suppl.e14574

Ding M, Lin J, Qin C, Fu Y, Du Y, Qiu X, Wei P, Xu T. Novel CAR-T Cells Specifically Targeting SIA-CIgG Demonstrate Effective Antitumor Efficacy in Bladder Cancer. Adv Sci (Weinh). 2024 Oct;11(40):e2400156. doi: 10.1002/advs.202400156. Epub 2024 Aug 23. PMID: 39178136; PMCID: PMC11516049.
